# Feasibility of tongue image detection for coronary artery disease: based on deep learning

**DOI:** 10.3389/fcvm.2024.1384977

**Published:** 2024-08-23

**Authors:** Mengyao Duan, Boyan Mao, Zijian Li, Chuhao Wang, Zhixi Hu, Jing Guan, Feng Li

**Affiliations:** ^1^ School of Traditional Chinese Medicine, Beijing University of Chinese Medicine, Beijing, China; ^2^ School of Life Science, Beijing University of Chinese Medicine, Beijing, China; ^3^ School of Traditional Chinese Medicine, Hunan University of Chinese Medicine, Changsha, China

**Keywords:** coronary artery disease, deep learning, hypertension, tongue image, early diagnosis

## Abstract

**Aim:**

Clarify the potential diagnostic value of tongue images for coronary artery disease (CAD), develop a CAD diagnostic model that enhances performance by incorporating tongue image inputs, and provide more reliable evidence for the clinical diagnosis of CAD, offering new biological characterization evidence.

**Methods:**

We recruited 684 patients from four hospitals in China for a cross-sectional study, collecting their baseline information and standardized tongue images to train and validate our CAD diagnostic algorithm. We used DeepLabV3 + for segmentation of the tongue body and employed Resnet-18, pretrained on ImageNet, to extract features from the tongue images. We applied DT (Decision Trees), RF (Random Forest), LR (Logistic Regression), SVM (Support Vector Machine), and XGBoost models, developing CAD diagnostic models with inputs of risk factors alone and then with the additional inclusion of tongue image features. We compared the diagnostic performance of different algorithms using accuracy, precision, recall, F1-score, AUPR, and AUC.

**Results:**

We classified patients with CAD using tongue images and found that this classification criterion was effective (ACC = 0.670, AUC = 0.690, Recall = 0.666). After comparing algorithms such as Decision Tree (DT), Random Forest (RF), Logistic Regression (LR), Support Vector Machine (SVM), and XGBoost, we ultimately chose XGBoost to develop the CAD diagnosis algorithm. The performance of the CAD diagnosis algorithm developed solely based on risk factors was ACC = 0.730, Precision = 0.811, AUC = 0.763. When tongue features were integrated, the performance of the CAD diagnosis algorithm improved to ACC = 0.760, Precision = 0.773, AUC = 0.786, Recall = 0.850, indicating an enhancement in performance.

**Conclusion:**

The use of tongue images in the diagnosis of CAD is feasible, and the inclusion of these features can enhance the performance of existing CAD diagnosis algorithms. We have customized this novel CAD diagnosis algorithm, which offers the advantages of being noninvasive, simple, and cost-effective. It is suitable for large-scale screening of CAD among hypertensive populations. Tongue image features may emerge as potential biomarkers and new risk indicators for CAD.

## 1 Introduction

The World Health Organization (WHO) has declared that cardiovascular disease (CVD), particularly coronary artery disease (CAD), is the leading cause of death due to illness globally, accounting for 17.9 million fatalities per year, representing 32% of all deaths caused by disease (1). This significant public health challenge places a significant financial burden on national health budgets (2). Hypertension is an important independent risk factor for the development of CAD (3). Relevant studies have shown that patients with hypertension have a higher risk of developing CAD, and when the two diseases coexist, there is a significant increase in the risk of cardiovascular death (4, 5). Early diagnosis and prompt treatment of patients with CAD have been shown to significantly improve outcomes and reduce treatment costs (3). However, the gold standard for diagnosing CAD is invasive coronary angiography, which is expensive and can cause complications (6). It is not suitable for early diagnosis and disease risk assessment. Finding non-invasive, cost-effective and efficient methods for early CAD diagnosis is crucial in global public health.

The rapid development of artificial intelligence in recent years has provided new insights into the exploration of non-invasive diagnostic methods for CAD. Clinical data exhibit complex and multidimensional characteristics, in which machine learning (ML) demonstrates advantages over traditional statistical methods (7). ML involves the selection and integration of multiple models. When confronted with the complex nonlinear relationships in clinical data, traditional statistical methods often struggle to accomplish modeling tasks. However, ML algorithms can automatically learn to handle nonlinear relationships and select useful predictive features. These algorithms can more effectively reveal hidden relationships within data and has been increasingly utilized for the diagnosis and risk prediction of clinical diseases.

Some scholars have already utilized ML to develop diagnostic models for CAD, with the predictive variables primarily being clinical risk factors (8–10). These studies demonstrate the promising application prospects of ML in clinical diagnostic tasks. Recent research has found that, in addition to risk factors, other biological information may also hold significant importance for the diagnosis of CAD, such as facial images and pulse waves (11, 12). In clinical diagnosis, the primary focus is on the patient's symptoms and signs.Traditional Chinese Medicine (TCM) employs unique and effective diagnostic strategies, particularly in observing the external conditions of patients. TCM theory posits that “internal diseases manifest externally,” thereby allowing practitioners to gauge the severity of illnesses through observation. Tongue diagnosis is a critical component of the TCM observation process. The appearance of the tongue, including its color, shape, and coating, has long been utilized in TCM to diagnose various health conditions. From a biomedical perspective, the tongue is a highly vascular organ closely related to the cardiovascular system. Changes in blood circulation and overall systemic health often manifest as observable alterations in the tongue's appearance. Many studies have proven the effectiveness of diagnosing diseases through tongue observation (references 14–27), and we have compiled these studies into Table 1 and provided commentary on each. From a biomedical standpoint, the tongue contains rich physiological and pathological information. It is an important terminal organ with abundant blood supply, closely linked to the cardiovascular system. When there are issues with blood circulation, the tongue's appearance often changes (27, 28). Biomedical research suggests that hypoxemia can lead to changes in tongue color and is associated with various cardiovascular diseases (29). In the case of CHD, the narrowing of the coronary arteries restricts blood flow to the heart, potentially causing systemic changes in overall circulation and oxygenation levels, which manifest on the tongue. Despite this, the tongue has not been effectively utilized in the actual diagnosis process of CAD. Recent advancements in artificial intelligence and deep learning have made it possible to extract and analyze subtle features from medical images, including tongue images, which were previously difficult to quantify. By leveraging these technologies, it is possible to detect patterns and features in tongue images associated with CAD, providing a non-invasive, cost-effective, and accessible diagnostic tool. Therefore, what exactly is the diagnostic value of the tongue for CAD? Can tongue images become a crucial basis for optimizing non-invasive diagnosis of CAD? This is precisely the question this study aims to explore.

**Table 1:** Research on tongue image-assisted disease diagnosis in the past 3 years.

Study name	Year	Authors	Main findings	Comments	Diagnosis method
Improvement in tongue color image analysis for disease identification using deep learning based depthwise separable (13)	2021	Rajakumaran S, Sasikala J	Deep learning-based depthwise separable method enhances disease identification through tongue color image analysis.	Novel method with potential applications, needs more research.	Deep Learning and Tongue Color Image Analysis
Application of computer tongue image analysis technology in the diagnosis of NAFLD (14)	2021	Jiang T, Guo X, Tu L, et al.	Computer tongue image analysis aids in non-invasive diagnosis of NAFLD.	Effective for NAFLD, could be adapted for heart disease diagnosis.	Computer Tongue Image Analysis
Internet of things and synergic deep learning based biomedical tongue color image analysis for disease diagnosis and classification (15)	2021	Mansour RF, Althobaiti MM, Ashour AA	IoT and synergic deep learning based tongue color image analysis improves disease diagnosis.	Combines IoT and deep learning for enhanced	Using IoT and synergistic deep learning for tongue image analysis
Establishment of noninvasive diabetes risk prediction model based on tongue features and machine learning techniques (16)	2021	Li J, Chen Q, Hu X, Yuan P,et al.	Noninvasive risk prediction model for diabetes based on tongue features and ML techniques.	Promising noninvasive risk prediction.	Noninvasive Risk Prediction Model based on tongue image
Intelligent deep learning based disease diagnosis using biomedical tongue images (17)	2022	Thanikachalam V, Shanthi S, Kalirajan K,et al	Deep learning-based analysis of biomedical tongue images aids in the diagnosis of multiple diseases including coronary heart disease.	Promising results but requires large-scale validation.	Deep Learning and Biomedical Tongue Images
Deep Learning Multi-label Tongue Image Analysis and Its Application in a Population Undergoing Routine Medical Checkup (18)	2022	Jiang T, Lu Z, Hu X,et al	Deep learning multi-label tongue image analysis is effective for routine medical checkups.	Tongue images based on deep learning can assist in medical examinations.	Multilabel tongue images processed based on deep learning
A multi-step approach for tongue image classification in patients with diabetes (19)	2022	Li J, Huang J, Jiang T, et al	Multi-step approach enhances tongue image classification for diabetes patients.	Effective for patient-specific diagnosis.	Multi-Step Classification Approach based on tongue image
Simulated Annealing with Deep Learning Based Tongue Image Analysis for Heart Disease Diagnosis (20)	2023	Sivasubramaniam S, Balamurugan SP	Tongue image analysis using deep learning and simulated annealing improves heart disease diagnosis.	Effective but needs further clinical validation.	Tongue Image Analysis and Deep Learning
Multiple color representation and fusion for diabetes mellitus diagnosis based on back tongue images (21)	2023	Zhang N, Jiang Z, Li JX, et al	Multiple color representation and fusion improve diabetes diagnosis based on tongue images.	Enhanced diabetes diagnosis with feature fusion.	Multicolor representation and fusion methods for processing tongue images
Development of a tongue image-based machine learning tool for the diagnosis of gastric cancer: a prospective multicentre clinical cohort study (22)	2023	Yuan L, Yang L, Zhang S, Xu Z, et al.	Tongue image-based machine learning tool developed for gastric cancer diagnosis in a multicentre clinical cohort study.	Validated in multi-center studies, this method shows potential for application to other diseases.	Tongue Image-Based Machine Learning Tool
Reliability of non-contact tongue diagnosis for Sjögren's syndrome using machine learning method (23)	2023	Noguchi K, Saito I, Namiki T, Yoshimura Y, et al.	Non-contact tongue diagnosis for Sjögren's syndrome is reliable using machine learning.	Tongue images can be used for contactless diagnosis of Sjögren's syndrome.	Tongue Diagnosis and machine learning
A lung cancer risk warning model based on tongue images (24)	2023	Shi Y, Guo D, Chun Y, et al.	Tongue image analysis can provide early warning for lung cancer risk.	Promising for early detection and risk assessment.	Tongue Image Analysis and Machine Learning
Application of intelligent tongue image analysis in Conjunction with microbiomes in the diagnosis of MAFLD (25)	2024	Dai S, Guo X, Liu S, et al.	Intelligent tongue image analysis combined with microbiome data improves diagnosis of MAFLD.	The potential of using the tongue for disease diagnosis based on deep learning has been demonstrated.	Intelligent Tongue Image Analysis and Microbiomes
Machine learning aided non-invasive diagnosis of coronary heart disease based on tongue features fusion (26)	2024	Duan M, Zhang Y, Liu Y, et al.	Tongue features fusion with machine learning aids non-invasive coronary heart disease diagnosis.	Feature fusion method improves diagnosis accuracy.	Tongue Features Fusion and Machine Learning

To this end, we conducted a multi-center cross-sectional clinical study, utilizing deep learning methods to explore the potential connection between tongue image features and CAD. Additionally, we aimed to investigate the feasibility of optimizing CAD diagnostic models by incorporating tongue image features as risk factors. Meanwhile, we also hope to present a new and effective biomarker for the clinical diagnosis of CAD.

## 2 Materials and methods

### 2.1 Study population and ethical statement

From March 2019 to November 2022, hypertensive patients aged 18–85 were recruited from the cardiology departments of Dongzhimen Hospital, Dongfang Hospital, the Third Affiliated Hospital of Beijing University of Chinese Medicine, and the First Affiliated Hospital of Hunan University of Chinese Medicine. All participants signed an informed consent form, and the study was conducted in accordance with the Declaration of Helsinki. The ethical review of this study was carried out and approved by the Institutional Review Board (IRB) of Shuguang Hospital affiliated with Shanghai University of Traditional Chinese Medicine (IRB number: 2018-626-55-01), with the clinical trial registration number ChiCTR1900026008. All source codes and data analyzed in this study can be obtained from the corresponding author upon reasonable request.

The diagnostic criteria for hypertension refer to the “Chinese Guidelines for the Prevention and Treatment of Hypertension,” which define hypertension as a systolic blood pressure ≥140 mmHg or diastolic blood pressure ≥90 mmHg, or currently undergoing treatment with antihypertensive medication (30). The diagnosis of CAD is based on the patient's CAG results, that is, a narrowing of the inner diameter of at least one of the coronary arteries (left anterior descending, left circumflex, right coronary artery, or left main) by ≥50%. Initially, 684 patients were recruited, with the following exclusion criteria: (1) patients whose tongue appearance was altered by medications or food, (2) no definitive diagnosis related to CAD, prior percutaneous coronary intervention (PCI) or coronary artery bypass grafting (CABG); (3) those with a severe lack of relevant clinical pathophysiological information; (4) those with poor quality tongue images.

### 2.2 Data collection

**Figure 1:**
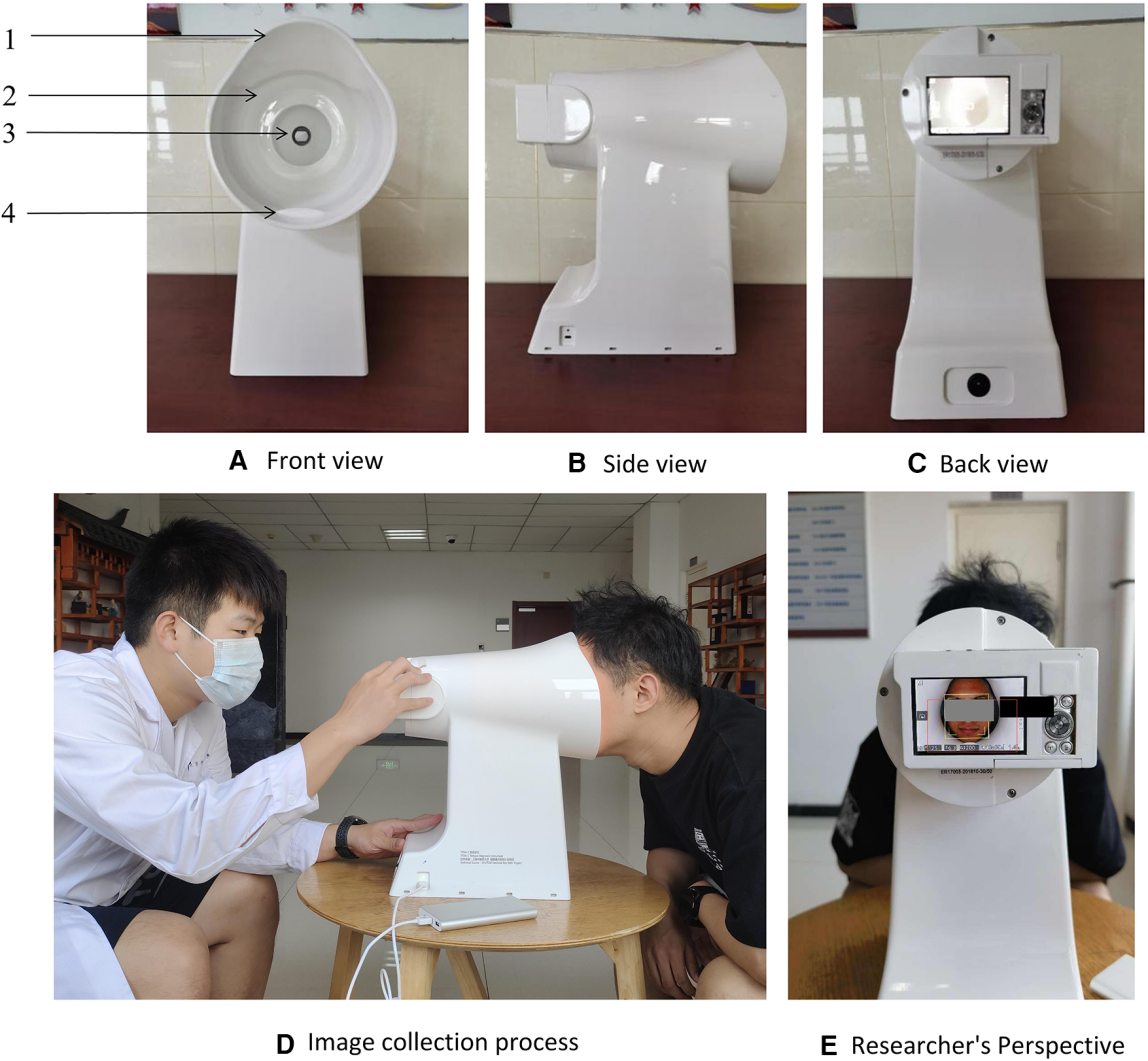
The tongue diagnosis instrument and collection process. 1: lens hood, 2: LED light resource, 3: high-definition camera, 4: chin support plate. Note. Use fixed standard camera parameters when shooting: the color temperature is 5,000 k, the color rendering index is 97, the frame rate is 1/125 s, the aperture is F/6.3, the exposure indicator scale is 0 or ±1. **(A)** Front view of the device; **(B)** Side view of the device; **(C)** Rear view of the device; **(D)** Schematic of the image acquisition process; **(E)** Perspective of the operator.

Trained research physicians conducted interviews and took tongue photographs of participants following a standardized collection process. The interviews gathered baseline data on general conditions, socioeconomic status, lifestyle (alcohol consumption, smoking, insomnia), and clinical manifestations. Tongue photographs were collected using a TFDA-1 tongue diagnosis instrument (Figure 1), two hours after breakfast or lunch. The specific steps for image collection are as follows: (1) Power on the Instrument after inspection and adjust the camera parameters. (2) Disinfect the areas of the instrument that may come into direct contact with the participant using 75% alcohol. (3) Instruct the patient to place their face on the chin rest, relax, and stick out their tongue flatly. (4) Turn on the built-in ring light source and complete the image capture. (5) Check the photo; if it is satisfactory, the collection is complete; if not, retake the photo until the image quality meets the standard. Qualification criteria for photo quality: no problems such as occlusion, blurring, fogging, overexposure, or underexposure; the tongue should be relaxed and flattened with no twisting or tension; there should be no foreign objects, staining, or other conditions affecting the appearance of the tongue surface.

### 2.3 Data preprocessing

Patients were divided into two groups based on whether they were diagnosed with CAD: the hypertension group and the hypertension combined with CAD group, with the labels recorded as 0 and 1, respectively.

**Figure 2:**
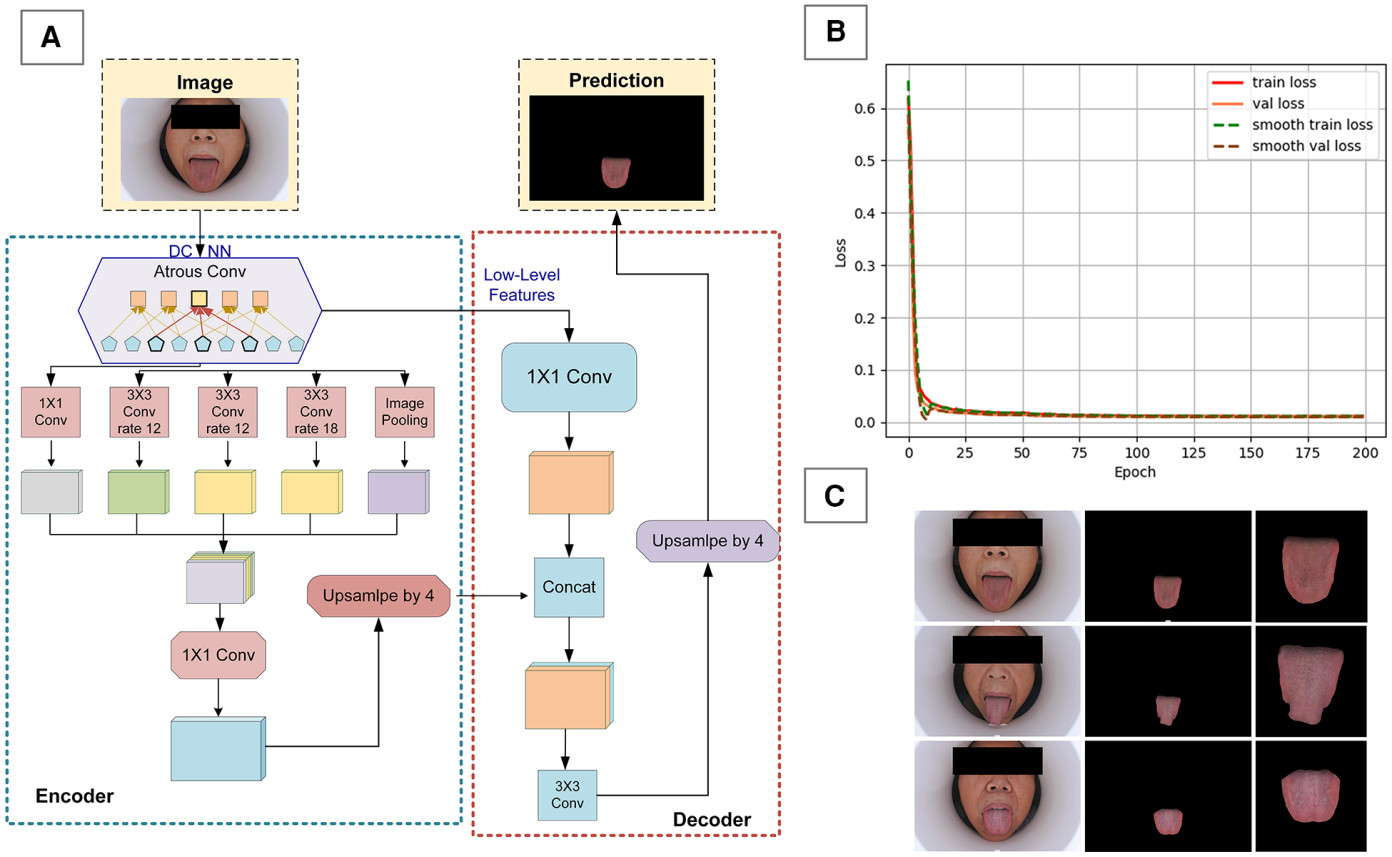
Data preprocessing for tongue images. **(A)** DeepLabV3 + framework diagram, **(B)** model training loss function graph, **(C)** preprocessing effect on tongue image.

Considering that tongue images also contain other facial information, which is superfluous for this study, we built a deep learning model for semantic segmentation of the tongue body using the DeepLabV3 + framework (Figure 2A). We used 500 images from a national key research and development program tongue image database for model training, implementing a phased training strategy. In the first 50 epochs of training (Figure 2B), the backbone of the model was frozen to focus on fine-tuning the tail end of the network, with a batch size set to 8. Subsequently, in the unfreezing phase, all network layers were involved in training, with the batch size adjusted to 4 and the learning rate set to 0.01. After completing the segmentation of the tongue body, the image size was uniformly cropped and adjusted to 256 × 256 pixels (Figure 2C). Additionally, due to our overall small sample size, data augmentation was performed on the images through rotation, flipping, and translation.

For the baseline data of patients obtained through interviews, there was only a minimal amount of missing data (less than 5% missing). Various interpolation methods were used to fill in the missing data. For discrete variables in the baseline data, such as gender and ethnicity, one-hot encoding was employed for data preprocessing.

### 2.4 Development of a CAD diagnostic algorithm

**Figure 3:**
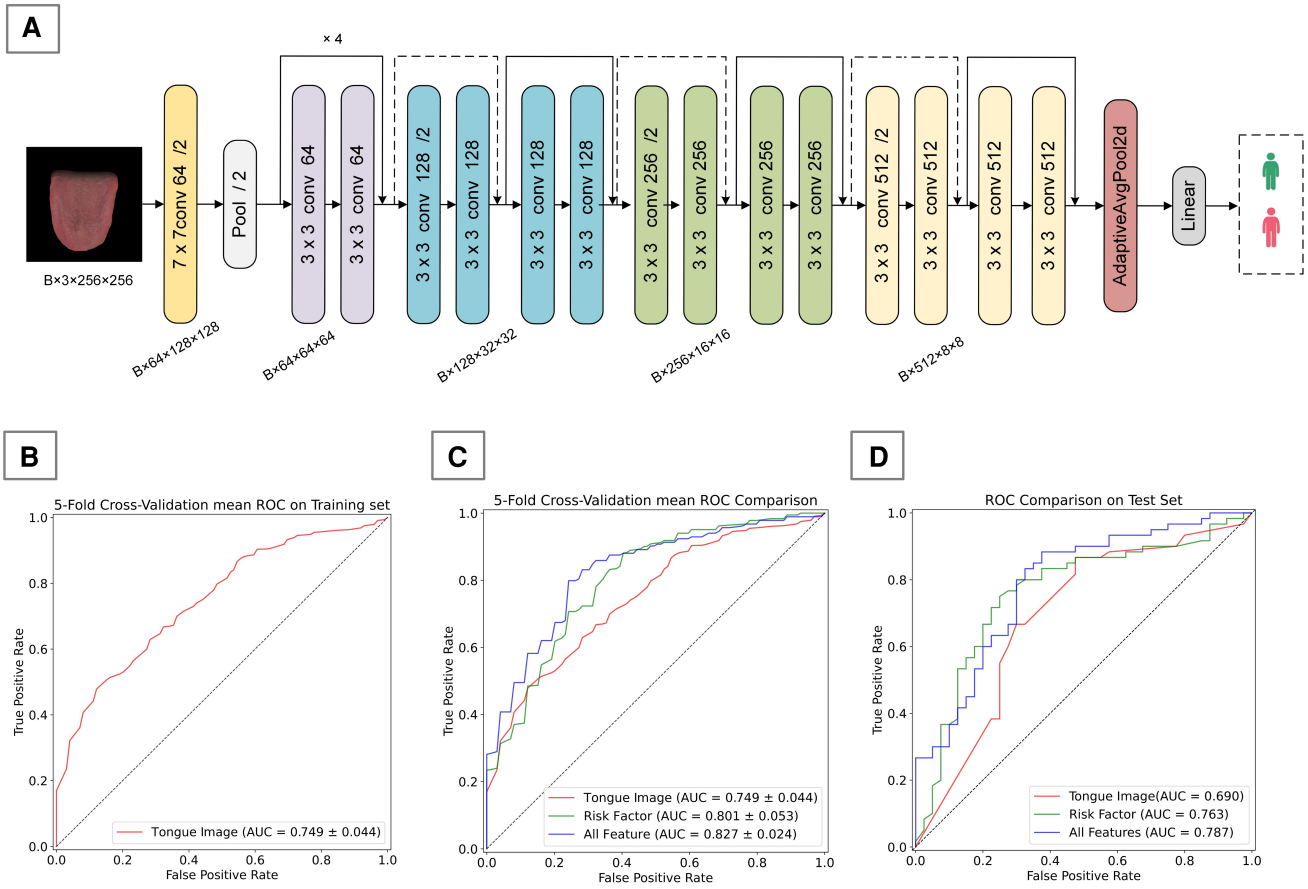
Tongue image-based CAD diagnostic algorithm. **(A)** ResNet-18 framework used in this study, **(B)** 5-fold cross-validation mean ROC on training set, **(C)** 5-fold cross-validation mean ROC Comparison on training set, **(D)** performance comparison of different feature inputs on the validation set.

The customization of a CAD diagnostic algorithm primarily encompasses two core steps: the classification of images using deep learning frameworks, and the construction of diagnostic models utilizing common ML techniques. In this study, we utilized the ResNet-18 network (Figure 3A), pretrained on the ImageNet dataset, as the foundation for our deep learning architecture. As a deep residual network, ResNet-18 effectively mitigates the vanishing gradient problem encountered in training deep networks through residual learning, making it widely applicable in image recognition, especially in the field of medical image processing. It has demonstrated excellent performance in various tongue image processing tasks.

During the training process, we chose to freeze the first and second layers (layer1 and layer2) of the model, training only the parameters of the third and fourth layers (layer3 and layer4) along with the fully connected layer. This strategy not only helps prevent overfitting, which might arise due to the small size of the dataset, but also further enhances the model's ability to learn representations. Stochastic Gradient Descent (SGD) was employed as the optimizer, with cross-entropy loss function used as the loss function. Upon convergence, the output of the penultimate layer of the model forms a 512-dimensional deep feature vector, which is then linked to a binary classification output layer to produce probabilities.

After extracting the deep feature vectors, we constructed a CAD diagnostic model that integrates tongue image feature vectors with risk factors. To optimize the model's performance, we explored a variety of common ML algorithms, including Random Forest (RF), Support Vector Machine (SVM), Decision Trees (DT), Logistic Regression (LR), and XGBoost, all of which are widely used in disease classification and risk prediction. By comparing the performance of these algorithms, we will select the one with the best performance as the core algorithm for our final CAD diagnostic model.

### 2.5 Statistical analysis

Using SPSS 27 and Python 3.8 as statistical tools, we processed and analyzed the data. For data that followed a normal distribution, we employed descriptive statistics using $\bar{X}\pm \mathrm{SD}$, along with one-way ANOVA to explore differences between groups. Before conducting the one-way ANOVA, we performed a homogeneity of variance test, such as Levene's test, to ensure that the variances across groups were roughly equal. For data not meeting the normal distribution criteria, we opted for quartile descriptions and employed the Kruskal–Wallis H test to compare differences between two groups. Additionally, we conducted an in-depth analysis of potential risk factors for CAD using binary logistic regression, presenting the results with adjusted odds ratios (adjusted OR) and their 95% confidence intervals (CI). Throughout the analysis, differences were considered statistically significant when the *p*-value < 0.05.

### 2.6 Performance evaluation standard

To evaluate the algorithm's performance, we calculated the Accuracy, Precision, Recall, F-1 Score, AUC (Area Under the Curve), and AUPR (Area Under the Precision-Recall Curve) (Equations 1–4) (31).

(1) $$\mathrm{Accuracy}=\frac{\mathrm{TP}+\mathrm{TN}}{\mathrm{TP}+\mathrm{FN}+\mathrm{FP}+\mathrm{TN}}$$

(2) $$\mathrm{Precision}=\frac{\mathrm{TP}}{\mathrm{TP}+\mathrm{FP}}$$

(3) $$\mathrm{Recall}=\frac{\mathrm{TP}}{\mathrm{TP}+\mathrm{FN}}$$

(4) $$F1=2\times \frac{\mathrm{Precision}\times \mathrm{Recall}}{\mathrm{Precision}+\mathrm{Recall}}$$

For binary classification problems, examples can be divided into four categories based on the combination of their true labels and the predictions made by the classifier: True Positives (TP), False Positives (FP), True Negatives (TN), and False Negatives (FN). In addition, we performed a 5-fold cross-validation on the training set to evaluate the model's effectiveness.

We evaluated the segmentation results of the DeepLabV3 + model using Pixel Accuracy (PA) and Mean Intersection over Union (MIoU) (Equations 5, 6). The formulas for calculating PA and MIoU are as follows, where *k* represents the number of categories excluding the background, and *p*_ij_ denotes the number of pixels of class *i* predicted to be class *j* (32):

(5) $$\mathrm{PA}=\frac{\sum_{i=0}^{k}\;p_{\mathrm{ii}}}{\sum_{i=0}^{k}\sum_{\;j=0}^{k}\;p_{\mathrm{ij}}}$$

(6) $$\mathrm{MIoU}=\frac{1}{k+1}\overset{k}{\underset{i=1}{\sum }}\frac{\;p_{\mathrm{ii}}}{\sum_{j=0}^{k}p_{\mathrm{ij}}+\sum_{j=0}^{k}p_{\mathrm{ji}}-p_{\mathrm{ii}}}$$

## 3 Results

### 3.1 Patient recruitment and data analysis

**Figure 4:**
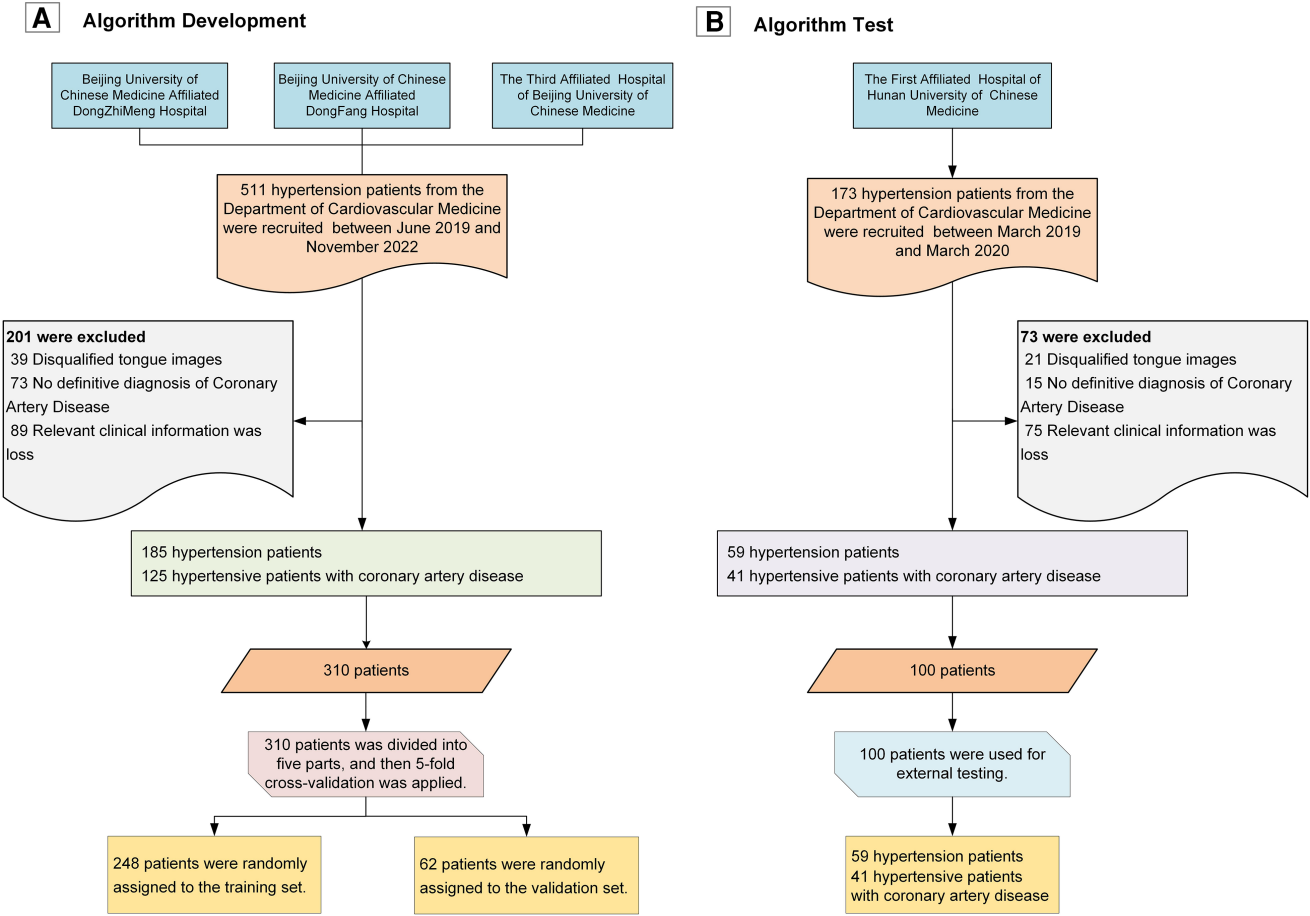
Study flowchart. **(A)** Workflow for algorithm development; **(B)** Workflow for algorithm validation.

As shown in Figure 4, we recruited a total of 511 hypertension patients from the cardiology departments of Dongzhimen Hospital, Dongfang Hospital, and the Third Affiliated Hospital of Beijing University of Chinese Medicine, and an additional 173 patients from the First Affiliated Hospital of Hunan University of Chinese Medicine. These patients underwent interviews and had standard tongue photographs taken. After screening, we excluded 60 patients (8.77%) with disqualifying tongue images, 164 patients (23.98%) with more than 5% missing data, and 88 patients (12.87%) without a coronary CAD record and who could not be definitively ruled out for CAD. Ultimately, we included a total of 244 hypertension patients and 166 patients with hypertension combined with CAD in our study.

Table 2 provides detailed baseline information for both patient groups. Upon analysis, we found significant differences between groups in terms of age, BMI, and the duration of hypertension across the training, validation, and test sets. To further explore potential risk factors, we analyzed the data from the training and validation sets using logistic regression (Table 3). The results indicated that older age, male gender, and having hyperlipidemia are risk factors for CAD, while having a higher education, Insomnia, and using antihypertensive medication were considered protective factors.

**Table 2:** Basic information of three groups.

Groups	*n*	Age	Male	BMI	Course of hypertension (year)
Training group					
Hypertension	149	66.51 (64.84–68.18)	62	25.97 ± 3.54	15.62 (13.63–17.60)
Hypertension with CAD	99	69.38 (67.14–71.63)	51	25.54 ± 4.39	15.71 (13.03–18.40)
Validation group					
Hypertension	37	68.14 (63.64–72.64)	21	24.18 ± 4.46	14.66 (11.12–18.21)
Hypertension with CAD	25	69.35 (63.99–71.71)	12	26.75 ± 3.60	18.29 (12.34–24.24)
Test group					
Hypertension	59	69.17 (64.33–71.22)	31	24.64 ± 3.67	9.12 (6.88–14.07)
Hypertension with CAD	41	69.62 (64.14–72.16)	21	25.34 ± 4.12	13.17 (7.36–17.85)
***P*-value**		**0.007**	0.399	**0.009**	**< 0.001**

Results with *P* < 0.05 are bolded.

**Table 3:** Binary logistic regression results of the main coronary artery disease risk factors.

Variables	Adjusted OR (95% CI)	*P-*value
Age	1.04 (1.01–1.06)	**0**.**009**
Male	2.24 (0.90–5.53)	**0**.**027**
BMI	1.06 (0.97–1.16)	0.194
Course of hypertension (year)	1.00 (0.98–1.02)	0.863
Education		
Less than high school	1.28 (0.63–2.62)	0.500
University	0.12 (0.03–1.06)	**0**.**004**
Lifestyle		
Alcohol(>2 times/week, >1 year)	0.59 (0.23–1.53)	0.279
Smoking(>2 times/day, >1 year)	0.54 (0.22–1.35)	0.187
Insomnia (>2 times/week)	0.89 (0.44–1.79)	**0**.**041**
Family history of CAD	1.63 (0.94–2.82)	0.079
Work status		
Work <4 h/day	0.53 (0.15–1.86)	0.317
4–10 h/day	0.76 (0.21–2.78)	0.676
>10 h/day	0.97 (0.45–2.46)	0.432
Hyperlipidemia	4.28 (1.92–9.50)	**<0.001**
Diabetes	0.72 (0.35–1.48)	0.377
Chronic renal disease	0.39 (0.14–1.10)	0.393
Antihypertensive medication	0.56 (0.32–1.01)	**0**.**049**

Results with *P* < 0.05 are bolded.

### 3.2 Performance of tongue feature extraction algorithm

In the task of semantic segmentation of the tongue body, we employed a customized algorithm based on the Deeplab V3 + framework, which demonstrated outstanding performance. The overall accuracy exceeded 99%, with a Mean Intersection over Union (mIoU) for the tongue segmentation task reaching 98.77%, and the Mean Pixel Accuracy (mPA) was as high as 99.45%. Such results fully attest to the effectiveness and accuracy of our algorithm in the task of tongue body semantic segmentation.

We developed a CAD diagnostic algorithm based on the ResNet-18 framework, which uses tongue images as input. On the training set, the algorithm showed a mean accuracy of 0.690, a mean AUC value of 0.749, and a mean recall rate of 0.842 (Table 4); on the test set, it achieved an accuracy of 0.670, an AUC value of 0.690, and a recall rate of 0.666 (Table 5). As shown in Figures 3A,C, the algorithm demonstrates certain classification capabilities on both the training and test sets, indicating that tongue images indeed possess classification value for the diagnosis of CAD in patients with hypertension.3.3 Performance of CAD Diagnostic Algorithm.

**Table 4:** Performance of 5-fold cross-validation with different feature inputs.

Input features	Mean accuracy	Mean precision	Mean recall	Mean F-1 score	Mean AUC	Mean AUPR
Tongue feature	0.690	0.699	0.842	0.763	0.749	0.807
Risk factors	0.761	0.778	0.837	0.806	0.801	0.842
Risk factors & tongue feature	0.794	0.795	0.886	0.836	0.827	0.865

**Table 5:** Performance on the test Set with different feature inputs.

Input features	Accuracy	Precision	Recall	F-1 score	AUC	AUPR
Tongue feature	0.670	0.754	0.666	0.708	0.690	0.717
Risk factors	0.730	0.811	0.717	0.761	0.763	0.800
Risk factors & tongue feature	0.760	0.773	0.850	0.809	0.786	0.845

We developed two types of CAD diagnostic models: one based solely on CAD risk factors and the other combining risk factors with deep features of tongue images. To determine the optimal ML approach, we compared the performance of various algorithms, all utilizing risk factors and deep features of tongue images as inputs. Results on the training set showed that different ML algorithms could effectively complete the classification task, with XGBoost exhibiting the best performance (Table 6). Therefore, we ultimately selected XGBoost as the method for algorithm customization. Moreover, we compared algorithm customized only with risk factors to those incorporating tongue image features, it was found that the inclusion of tongue image features significantly enhanced algorithm performance, indicating that adding tongue features as input variables positively contributes to algorithm optimization (Figure 3B; Table 4).

**Table 6:** Comparison of 5-fold cross-validation results across different algorithms.

Algorithm	Mean accuracy	Mean precision	Mean recall	Mean F-1 score	Mean AUC	Mean AUPR
DT	0.745	0.802	0.778	0.783	0.760	0.791
RF	0.777	0.793	0.848	0.817	0.818	0.851
SVM	0.742	0.753	0.859	0.799	0.799	0.856
LR	0.761	0.786	0.826	0.804	0.817	0.851
XGBoost	0.794	0.795	0.886	0.836	0.825	0.865

We also evaluated the performance of different ML algorithms on the test set (Table 7), and the results were broadly consistent with those on the validation set. Although there was a slight decrease in performance, the algorithms still demonstrated good classification capabilities, with XGBoost continuing to show the best performance. Additionally, we compared algorithms developed solely based on risk factors with those that integrate both risk factors and tongue image features, using the test set for evaluation (Figure 3C; Table 5). This finding confirms the practical diagnostic value of tongue images for CAD and also indicates the potential of tongue images to enhance the efficacy of current diagnostic models for the condition.

**Table 7:** The performance comparison of different algorithm on the test set.

Algorithm	Accuracy	Precision	Recall	F-1 score	AUC	AUPR
DT	0.710	0.738	0.800	0.768	0.708	0.785
RF	0.740	0.774	0.800	0.786	0.781	0.843
SVM	0.720	0.742	0.793	0.767	0.742	0.790
LR	0.750	0.769	0.833	0.800	0.751	0.823
XGBoost	0.760	0.773	0.850	0.809	0.786	0.845

**Figure 5:**
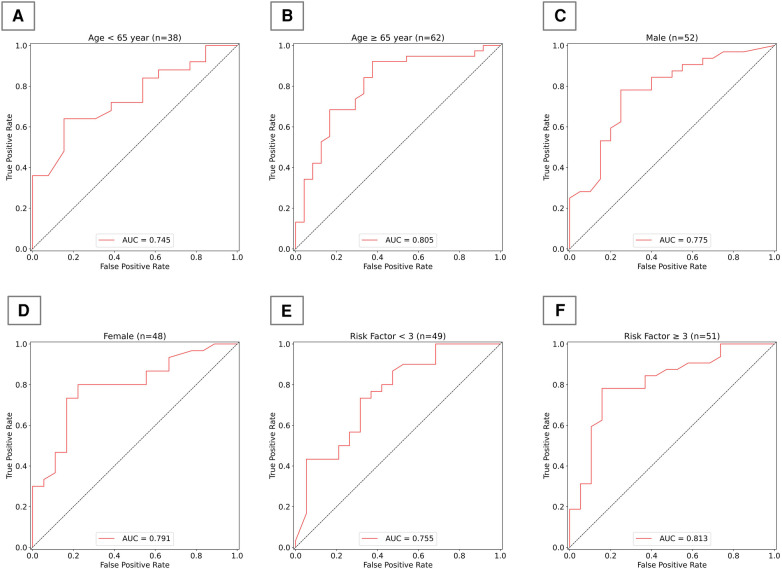
Algorithm performance in subgroups of test group. **(A)** AUC for the model in individuals under 65 years old; **(B)** AUC for the model in individuals 65 years and older; **(C)** AUC for the model in males; **(D)** AUC for the model in females; **(E)** AUC for the model in individuals with fewer than 3 risk factors; **(F)** AUC for the model in individuals with 3 or more risk factors.

To more comprehensively evaluate the algorithm's applicability and performance across different populations, we subdivided the test set according to age, gender, and the number of risk factors, and presented the algorithm's performance across various subgroups. As shown in Figure 5, in terms of age distribution, the algorithm demonstrated superior diagnostic ability in the elderly population aged 65 and above. Regarding gender, our algorithm demonstrated relatively stable performance between men and women, with no significant differences. In terms of risk factors, the algorithm's judgment ability significantly improved when the number of risk factors reached or exceeded three; however, with fewer risk factors, the algorithm's performance was comparatively weaker.

## 4 Discussion

Hypertensive patients constitute a large patient population and are an important risk factor for coronary heart disease. When the two conditions occur simultaneously, they can result in a higher burden of disease and accidental risks (4). Therefore, this study selected hypertensive patients as the target population. Tongue diagnosis is an important diagnostic method in TCM, closely related to the cardiovascular blood flow status. However, it has been largely overlooked in the actual diagnosis of CAD. In recent years, there have been many studies on constructing artificial intelligence diagnostic models for CAD, but none have attempted to utilize the tongue, a biological marker of the human body. We conducted a multi-center cross-sectional study, customizing a CAD diagnostic algorithm that integrates risk factors and tongue features based on clinically accessible data, achieving moderate performance. We innovated a method using deep learning to optimize CAD diagnosis through tongue images. This method has the advantages of being non-invasive, low-cost, and easy to operate compared to coronary angiography, and it exhibits better diagnostic performance than traditional CAD diagnostic algorithms that use risk factors. Our results affirm the practical diagnostic value of the tongue for CAD and demonstrate the feasibility of enhancing CAD diagnostic algorithm performance with tongue images.

For the development of a CAD diagnostic model, the rational use of clinical risk factors is of great importance. Various CAD diagnostic and prediction models, such as the Framingham (33) and the Systematic Coronary Risk Evaluation (SCORE) (34) heavily rely on clinical risk factors as a crucial basis. Inspired by this approach, our study first analyzed potential clinical risk factors for CAD. In our dataset, not all known CAD risk factors played a risk role, and insomnia, previously considered a risk factor (35) acted as a protective factor. We developed a CAD diagnostic algorithm that uses clinical risk factors as inputs, which exhibited moderate diagnostic performance (ACC = 0.770). Diagnostic models developed in other studies based on clinical risk factors showed similar performance (36–38). Although the performance of our non-invasive diagnostic model still needs further improvement, which may be related to the grouping method, sample size, and model construction approach, our results still demonstrate the potential of using tongue images for non-invasive diagnosis of CAD. Based on this, we attempted to incorporate tongue image features as inputs to develop a new CAD diagnostic algorithm. To accurately and objectively explore the value of tongue images for CAD diagnosis, we used the TFDA-1 tongue diagnosis instrument for image capture and established a standardized data collection process. We eliminated potential interference from other facial information through tongue body semantic segmentation and image cropping. In customizing the algorithm for diagnosing CAD with tongue images, we employed the Resnet-18 as the deep learning framework. Resnet-18 is a type of deep residual network (39) that has consistently shown good performance in past studies on tongue images (40–42). In the analysis of tongue image features using traditional feature engineering, medical prior knowledge is often borrowed to define features based on the color, shape, and texture of the tongue, ensuring that the features possess good interpretability and medical significance.

That is precisely why our research opted for deep learning instead of traditional feature engineering. Despite the complexity and lack of explainability in the decision-making process of deep learning, this method simplifies the feature extraction process compared to traditional tongue image feature extraction engineering (43). It eliminates the need for extensive manual labeling of image features and enables fast and efficient automatic learning of complex feature representations in images, uncovering hidden information. The results indicate that, although there was a slight decline in the algorithm's performance on the test set compared to the validation set, the algorithm still retains certain classification capabilities overall. This suggests that tongue images have a definite diagnostic value for CAD, making the tongue an effective biological marker for CAD diagnosis.

Ultimately, we incorporated both risk factors and tongue image features as inputs and developed a new CAD diagnostic algorithm using XGBoost. This algorithm demonstrates superior performance compared to those utilizing single-type features as inputs. This part of the work validates the practical effectiveness of tongue images in enhancing the performance of CAD diagnostic algorithms, proving the feasibility of supplementing clinical diagnosis with TCM diagnostic theories. It offers a new perspective on integrating traditional medical knowledge with modern technology. Additionally, we also focused on the algorithm's performance across different demographic subgroups. The results indicate that the algorithm has better diagnostic capability in elderly populations aged 65 years and above, which may be related to the higher prevalence of CAD in older individuals. The algorithm performs similarly in both men and women, indicating that our developed algorithm has commendable generalization capability across genders. The model exhibits higher accuracy in judgments when the number of risk factors is three or more, highlighting the importance of considering multiple risk factors in the diagnosis of CAD. This approach enhances our understanding of the model's generalizability, revealing its applicability to patient groups with varying demographic and clinical profiles. It holds significant value for clinical practice, offering a reference for tailoring diagnostic methods based on demographic characteristics to improve diagnostic accuracy. By evaluating the model's performance in different subgroups, we can identify potential biases that might affect accuracy, thereby rendering it more reliable in clinical settings.

Although this study identified the potential value of the tongue in diagnosing CAD, it also has some limitations. Firstly, despite being a multi-center study, there are only four hospitals from two regions involved in the sub-centers, lacking subjects from different ethnicities and countries. Secondly, this study is focused solely on hypertensive populations, and the overall sample size is relatively small, which may limit the possibility of applying the CAD diagnostic algorithm to a wider population. Thirdly, while this study employed standardized tongue image collection equipment to minimize interference from other factors during image capture, it also restricts the application of the model in different scenarios and with different collection devices. Although this study explores the potential value of tongue diagnosis for CAD, future research needs to further validate and optimize our diagnostic model in a wider and larger population, carrying out prospective studies. We also experimented with using different types of cameras, various light sources, and even mobile portable devices for image collection, to further expand the model's applicability and enhance its generalization capability. In future research, we can expect more optimized and interpretable deep learning models to enhance the study results, capturing finer changes in tongue images more accurately, thus further optimizing the findings of this study. Additionally, tongue diagnosis is only an essential component of TCM diagnosis, and we can further focus on the integration of multimodal data, considering the fusion of other biomarkers with tongue images to build a more comprehensive and integrated CAD diagnostic model.

## 5 Conclusion

Exploring an inexpensive, non-invasive diagnostic tool that can be used for early-stage and large-scale screening of CAD is essential. In this study, we analyzed potential risk factors for CAD, extracted potential diagnostic features from tongue images, and developed a new, well-performing CAD diagnostic algorithm based on these findings. Our work introduces a novel perspective, suggesting that tongue images have applicable diagnostic value for CAD diagnosis. Tongue image features could become new risk indicators for CAD, demonstrating the feasibility of integrating TCM theories with modern technology.

## Data Availability

The raw data supporting the conclusions of this article will be made available by the authors, without undue reservation.
